# Essential and Toxic Elements in Cardiovascular Disease: Pathophysiological Roles and the Emerging Contribution of Hair Mineral Analysis

**DOI:** 10.3390/ijms262412145

**Published:** 2025-12-17

**Authors:** Zofia Gramala, Oliwia Kalus, Joanna Maćkowiak, Katarzyna Zalewska, Michał Karpiński, Antoni Staniewski, Zofia Szymańska, Maciej Zieliński, Malwina Grobelna, Paweł Zawadzki, Ryszard Staniszewski, Aleksandra Krasińska-Płachta, Paulina Mertowska, Mansur Rahnama-Hezavah, Ewelina Grywalska, Tomasz Urbanowicz

**Affiliations:** 1 Students’ Cardiology Research Group, Poznan University of Medical Sciences, 61-701 Poznan, Poland; 2 Department of Vascular, Endovascular Surgery, Angiology and Phlebology, Poznan University of Medical Science, 61-848 Poznan, Poland; 3 Department of Ophthalmology, Poznan University of Medical Sciences, 61-107 Poznan, Poland; 4 Department of Experimental Immunology, Medical University of Lublin, 20-093 Lublin, Poland; 5 Department of Dental Surgery, Medical University of Lublin, 20-093 Lublin, Poland; 6 Cardiac Surgery and Transplantology Department, Poznan University of Medical Sciences, 61-848 Poznan, Poland

**Keywords:** hair mineral analysis, cardiovascular disease, trace elements, toxic metals, ICP-MS, environmental exposure, oxidative stress, biomonitoring, micronutrients, atherosclerosis

## Abstract

Hair mineral analysis (HMA) has emerged as a promising non-invasive method for assessing long-term exposure to trace elements and metals, potentially complementing traditional biochemical and clinical markers of cardiovascular risk. This review synthesizes current evidence on the relationships between hair elemental profiles and cardiovascular disease (CVD), with an emphasis on toxic metals (As, Hg, Pb, Cd, Ni, Al) and essential micronutrients (Mg, Mn, Zn, Cu, Fe, Cr, Li). The reviewed studies consistently show that patients with CVD exhibit elevated levels of toxic elements and reduced concentrations of protective ones, reflecting oxidative stress, inflammation, and endothelial dysfunction as mechanistic links. Methodologically, the review highlights inductively coupled plasma mass spectrometry (ICP-MS) with collision/reaction cell technology and microwave digestion as gold-standard analytical approaches, while underscoring the urgent need for harmonized protocols, validated washing procedures, and certified reference materials. The interpretation of HMA requires consideration of temporal dynamics, external contamination, and regional variability. Although current evidence supports the research utility of HMA, its clinical integration remains limited by the absence of reference ranges and prospective validation. HMA may hold future value in environmental risk stratification and primary prevention in exposed populations, but further standardization and large-scale longitudinal studies are necessary to define its diagnostic and prognostic relevance in cardiovascular medicine.

## 1. Introduction

Cardiovascular diseases (CVDs) have been the leading cause of morbidity and mortality globally for decades [1,2]. In 2020, circulatory system diseases—primarily heart disease and stroke—were responsible for more deaths than cancer and chronic lower respiratory tract diseases [3]. The number of people living with CVD increased from 271 million in 1990 to 523 million in 2019, and the annual number of deaths increased from 12.1 million to approximately 18.6–19 million (~32% of all deaths). The most significant burden falls on low- and middle-income populations, where limited access to prevention and treatment further exacerbates the problem [4]. Clinically, myocardial infarction, stroke, and arterial hypertension predominate [4,5,6], the basis of which is largely atherosclerosis—a chronic inflammatory and lipid process leading to arterial narrowing (ASCVD) [7,8,9].

About 70% of CVD cases and deaths are associated with modifiable factors—metabolic (diabetes, hypertension, obesity, insulin resistance), behavioral (psychosocial stress), and environmental, including air pollution, unhealthy diet, and low physical fitness [10,11,12]. The role of exposure to metals (aluminum, copper, lead, arsenic, cadmium) has also been increasingly documented, which increases the risk of CVD through cardiotoxicity, oxidative stress, and metabolic disorders [13,14,15]. The efficiency of antioxidant systems, particularly those involving selenium-dependent glutathione peroxidase (GPx), may mitigate the effects of these exposures and aging-related processes in the circulatory system [16]. At the same time, the balance of essential micro- and macronutrients (including calcium, vitamin D, zinc, and selenium) remains crucial for cardiovascular homeostasis, with both deficiency and excess potentially causing adverse effects [17].

Against this backdrop, diagnostic tools that capture the long-term “imprint” of elemental exposure are gaining importance. From an analytical perspective, hair provides a robust long-term matrix for selected elements that strongly bind to keratin and accumulate over time—in particular, toxic metals such as cadmium, lead, arsenic, mercury, nickel, and aluminum, as well as several essential trace elements including zinc, copper, selenium, and manganese. In contrast, for rapidly exchanging electrolytes (e.g., sodium and potassium) the correlations between hair content and systemic status are weaker and less well validated. Although the standard relies on blood analyses, hair mineral analysis (HMA) is a promising, non-invasive approach. Hair accumulates metals and trace elements during growth, providing a window of exposure of weeks to months—potentially useful for risk stratification, early detection of atherosclerotic lesions, and monitoring the effectiveness of nutritional and environmental interventions [18,19]. Reports indicate that the profile of hair elements may correlate with the location and severity of vascular lesions, broadening our understanding of the pathophysiology of CVD [20]. At the same time, a critical evaluation of the method is necessary: standardization of collection (site, strand length), sample washing and preparation procedures, analysis (preferably ICP-MS), control of external contamination, and interpretation of results in the context of age, gender, diet, smoking, medications, and comorbidities. Only then can HMA become a reliable complement to classic biomarkers [19,21].

The purpose of this narrative review article is to provide an HMA-focused evidence synthesis and critically evaluate the utility of hair trace element analysis in the context of cardiovascular disease. Our goal is to address the clinically relevant question of whether and how hair trace element profiles can become a reliable, noninvasive component of risk assessment, early detection, and monitoring of cardiovascular disease, and what standards must be met to incorporate HMA into evidence-based practice.

## 2. The Importance of Macronutrients and Micronutrients in Cardiovascular Disease

Macronutrients and micronutrients are essential for cardiovascular homeostasis. They modulate hemodynamics, myocardial excitability, cellular energy metabolism, oxidative stress, inflammation, and vascular mineralization, thereby influencing both the development and the clinical course of CVD. In routine cardiology, a relatively small group of elements accounts for the majority of well-documented clinical effects: among macronutrients, sodium, potassium, magnesium, calcium, and phosphate, and among micronutrients, iron, zinc, copper, and selenium [22,23,24,25,26].

Sodium and phosphate primarily affect circulating blood volume, vascular stiffness, and vascular/valvular calcification. Potassium, magnesium, and calcium play a crucial role in determining cardiac electrophysiology and the risk of arrhythmias, while magnesium also influences vascular tone and blood pressure control. Iron, zinc, copper, and selenium are key components of mitochondrial enzymes and antioxidant systems (including superoxide dismutases and selenoproteins), shaping redox balance, endothelial function, lipid peroxidation, and low-grade inflammation. Disturbances in the levels of these elements—either deficiency or excess—have been consistently linked to an increased risk of hypertension, coronary artery disease, heart failure, stroke and sudden cardiac death [22,23,24,25,26].

From a clinical standpoint, it is therefore crucial to maintain element concentrations within physiological ranges and to correct documented abnormalities (e.g., intravenous iron in iron-deficient heart failure, magnesium supplementation in torsade de pointes, control of calcium–phosphate imbalance in chronic kidney disease). Table 1 and Table 2 summarize their principal physiological roles, cardiovascular effects, and clinical implications.

Interactions between macro- and micronutrients and cardiac pharmacotherapy are frequent, often additive, and—importantly—predictable. By modulating blood volume and RAA activation, sodium can override the efficacy of antihypertensive drugs, potassium and magnesium directly determine the safety of antiarrhythmic therapy, and calcium critically interacts with digoxin and CCBs. Among micronutrients, iron deficiency impairs performance in HF, zinc and copper affect endothelial redox (and each other), selenium is crucial for antioxidant and thyroid defense (with significant hemodynamic consequences), and manganese, chromium, iodine, and phosphorus modify indirect metabolic pathways and vascular mineralization (Supplementary Materials Tables S1 and S2).

## 3. Hair Trace Element Analysis

Hair trace element analysis (HAA) is a valuable long-term biomonitoring tool. It is used to assess chronic exposure to toxic metals and to assess nutritional status in terms of micro- and macronutrients [65,66,67]. Hair grows on average about 1 cm per month, therefore providing a temporal archive of exposure over the past weeks and months. This makes HAA results less sensitive to short-term fluctuations, which are typical of blood or urine. Elements are incorporated into hair endogenously during keratinization of the hair shaft and can also be deposited exogenously from the environment, sweat, and sebum. Reliable interpretation requires distinguishing between these two pathways [66,67,68].

The reliability of results depends on the strict standardization of preanalytical steps. It is recommended to collect several strands from the occipital region, close to the skin, and analyze only a 3–4 cm section from the root. This segment reflects the average mineral metabolism over the last 3–4 months [69,70]. The test material should be clean and free of styling agents. For color-treated hair, it is recommended to wait at least 3–4 cm of hair to grow back or postpone collection for 6–8 weeks to minimize the impact of cosmetic treatments on the results [65,71]. A validated washing procedure that removes external contaminants without rinsing out the endogenous fraction is also crucial.

Highly sensitive techniques are used for elemental analysis in the keratin matrix. Inductively coupled plasma mass spectrometry (ICP-MS), often with a collision-reaction cell to reduce polyatomic interferences, is considered the first-choice method [72,73,74,75]. The use of microwave digestion in mineralization mixtures improves recovery and reproducibility. Instrumental advances in ICP-MS and sample preparation have increased precision, accuracy, reliability, and lowered detection limits [75,76]. Quality assurance encompasses the use of certified reference materials, participation in proficiency testing, reporting measurement uncertainty, and cleaning validation, as there is no single universal procedure for removing external contamination [70,71,76].

Interpretation of results must take into account the method’s time horizon. The HMA reflects a portion of mineral metabolism over recent months and does not describe an acute clinical condition. The analysis should include both absolute concentrations and element ratios, such as Ca/Mg, Na/K, or Zn/Cu. These indicators reflect the biochemical balance essential for maintaining homeostasis. In selected situations, segmented longitudinal hair shaft analysis is functional, as it enables the correlation of concentration changes over time and is useful in tracking chronic exposures of varying intensities [77,78,79]. The greatest challenge remains the lack of widespread standardization of procedures and the limited availability of reference values adapted to biological and environmental variability. The literature emphasizes that the HMA can be the subject of methodological disputes. Discussions concern both analytical accuracy and the lack of uniform treatment protocols. A research priority is to establish interoperable procedures for collection, washing, digestion, calibration, and quality control, as well as to develop reliable reference ranges that take into account age, gender, hair type, environmental factors, and regional differences. Until standards are fully harmonized, HMA results should be interpreted in triangulation with other data sources, such as blood and urine assays, detailed exposure history, and environmental information, while maintaining complete transparency of the methodology and analytical quality parameters [77,80,81,82].

## 4. The Role of Hair Trace Element Analysis in the Context of Cardiovascular Disease

### 4.1. Selenium

Selenium in the context of CVD is often assessed using hair trace element analysis (HMA), which reflects long-term body mineral status. A case–control study of 81 individuals with hyperlipidemia (types IIa/IIb) and 43 healthy volunteers found significantly higher hair selenium concentrations in patients with lipid disorders than in the controls, suggesting a link between selenium status and an unfavorable lipid profile. Under extremely deficient conditions—classically in Keshan disease—populations from endemic areas were characterized by very low hair selenium levels (<0.12 ppm), which correlated with the incidence of cardiomyopathy and has become a practical risk marker in these regions [83,84]. Biologically, selenium acts primarily through selenoproteins (e.g., glutathione peroxidase), which limit lipid peroxidation and oxidative damage to the heart muscle. Se deficiency therefore increases oxidative stress, impairs endothelial protection, and may promote the development of heart failure. In contrast, a moderate, physiological selenium status is considered cardioprotective [85]. At the same time, the results of interventions are not precise: reviews and meta-analyses of randomized trials do not confirm that selenium supplementation alone reduces the risk of CVD hard endpoints in populations without overt deficiency, which indicates that high Se levels (also recorded in hair) do not guarantee clinical benefits [85]. From a cardiovascular standpoint, hair selenium is therefore best regarded as an indicator of long-term Se exposure in epidemiological or high-exposure settings. Decisions on supplementation and clinical management should be guided primarily by systemic biomarkers and thyroid and lipid status [86,87].

### 4.2. Zinc

Zinc (Zn) plays a multifaceted role in the pathophysiology of the cardiovascular system, serving as a cofactor for Cu/Zn-SOD, a regulator of lipid and carbohydrate metabolism, and a modulator of the inflammatory response. Its deficiency may promote oxidative stress, atherosclerosis, and hypertension. However, the results of HMA remain inconsistent across populations. Some studies—particularly those related to myocardial infarction—have indicated reduced hair Zn levels compared to controls, which has been interpreted as a marker of long-term deficiency (integration time ~6–8 weeks) [83,88,89]. On the other hand, the study by Dziedzic et al. (2022) conducted in patients with angiographically confirmed coronary artery disease showed neither differences in hair Zn concentrations between patients and controls, nor an association with disease severity or acute vs. stable phenotype, questioning the usefulness of a single hair Zn measurement as a marker of CAD risk [90]. Importantly, lower hair Zn concentrations are observed in populations with metabolic disorders—e.g., in obese women (with and without hypertension) compared to healthy ones—suggesting that the hair signal may be strongly influenced by coexisting metabolic phenotypes and not solely by CVD per se [91,92]. At the mechanistic level, the importance of the Zn/Cu ratio has long been debated: the classical Klevaya hypothesis assumes that an imbalance of these elements may promote coronary heart disease, and newer approaches update this concept, emphasizing the relationship (ratio) rather than the absolute values of individual trace elements [93]. In systemic biomarkers, the data are more consistent: meta-analyses more often show an association between low serum Zn concentration and the presence/severity of CAD, which strengthens the preference for serum assessment in clinical practice, while HMA is more likely to be found in population-based or exploratory studies [94].

A zinc-specific methodological issue is the risk of external contamination from cosmetic products: residues of zinc-containing shampoos (e.g., zinc pyrithione) may remain on the hair shaft despite decontamination and artificially increase measured Zn levels [95,96,97,98]. Therefore, hair zinc should be interpreted in conjunction with information on cosmetic use and preferably in terms of Zn/Cu balance rather than as an isolated absolute value [90].

### 4.3. Copper

Copper is a key trace element for antioxidant and tissue enzymes (including Cu/Zn-SOD, ceruloplasmin, and lysyl oxidase), and its disturbances can modulate oxidative stress, inflammation, vessel wall remodeling, and iron metabolism—mechanisms important in atherosclerosis and hypertension. An excess of circulating “free” copper promotes free radical reactivity, while a deficiency weakens enzymatic systems. Its relationship with zinc (Cu/Zn ratio) is also essential as an indicator of redox balance and inflammation [99].

HMA studies have shown equivocal results regarding Cu in CVD. In the study by Dziedzic et al. (2022), in patients undergoing angiography due to suspected ACS, neither hair Cu content nor the Cu/Zn ratio was found to be associated with the occurrence of ACS or classical CAD risk factors, which argues against the diagnostic utility of a single hair Cu measurement in acute clinical situations [100]. In the study by Dziedzic et al. (2023), in patients with confirmed CAD, an inverse relationship was found between the severity of lesions (SYNTAX) and hair Cu content and the Cu/Zn ratio, with no association for Zn alone, suggesting that the “copper” signal may better reflect the complexity of atherosclerotic lesions than zinc assessed in isolation [89]. Differences between populations and metabolic phenotypes are significant. In a 2023 analysis, obese women had lower hair Cu concentrations. In contrast, hypertensive individuals had higher concentrations, regardless of body weight, suggesting a modulating effect of obesity and hypertension on hair Cu deposition [91].

Higher-certainty evidence comes from systemic biomarkers: meta-analyses and cohort analyses indicate that elevated plasma/serum Cu concentrations are associated with an increased risk of MACE, stroke, CAD, and cardiovascular mortality—supporting the pathophysiological role of copper as a risk factor in excess [101,102,103]. Mechanistically, this is linked to copper transport and homeostasis (ATP7A/ATP7B, ceruloplasmin, Atox1), as well as pro- and antioxidant effects, which depend on the element’s pool and location [99].

A copper-specific problem in HMA is cosmetic interference: hair dyeing can significantly increase measured Cu concentrations even after standard washing procedures, reducing analytical specificity. For this reason, information on hair cosmetic treatments is essential when interpreting hair copper and Cu/Zn ratios in CVD-related studies, while systemic Cu status is still assessed primarily using blood biomarkers.

### 4.4. Cadmium

Cadmium is a toxic heavy metal with no physiological function; its sources of exposure are primarily tobacco smoke and environmental pollutants. In HMA studies, higher Cd concentrations are typically observed in cardiac patients compared to healthy individuals. For example, in a classic Pakistani study of post-myocardial infarction patients, Cd levels in hair were significantly higher compared to controls, with a similar effect in both sexes [104]. The results of the international study by Skalny et al. (2021) also indicate that patients with CAD, especially obese individuals, exhibit elevated Cd content in their hair, with the highest values observed in obese individuals with CHD [105]. Not all studies confirm these differences (e.g., a 2015 study found no significant difference in Cd levels between MI and control groups, which was attributed to high environmental exposure in both groups); however, the overall trend remains consistent, with greater Cd accumulation in patients with CVD [106].

Mechanistically, Cd exhibits proatherogenic properties, as it damages the endothelium, increases inflammation and oxidative stress, and disrupts renal function, while also modulating the renin-angiotensin system, thereby promoting the development of hypertension and atherosclerosis [107]. Epidemiological data (in blood, urine, and hair) support a positive association between Cd exposure and the risk of hypertension and cardiovascular events, with more recent meta-analyses demonstrating an increasing risk of HT with exposure dose [108,109]. Cd may also impair the effect of protective elements (especially Zn and Se), which secondarily impairs redox balance and the anti-inflammatory response [110]. Importantly for population-based prevention, chronic maternal exposure to Cd (assessed in hair) has been associated with an increased risk of severe congenital heart defects in offspring [111,112].

From a biomarker perspective, hair cadmium is useful mainly for reconstructing long-term exposure and for public health screening of highly exposed populations (e.g., smokers, residents of industrial areas, occupationally exposed workers). Given the toxic potential of Cd, the key cardiovascular intervention remains reduction of exposure (smoking cessation, dietary and environmental control). At the same time, blood and urine Cd and organ damage markers (e.g., kidney function, blood pressure) guide clinical management.

### 4.5. Lead

Lead is highly cardiotoxic: it increases oxidative stress, impairs NO bioavailability and endothelial function, disrupts calcium signaling in vascular smooth muscle, and stresses the kidneys, promoting hypertension and the progression of atherosclerosis. These associations are supported by contemporary reviews and population analyses, including data indicating an association of even low-level Pb exposure with a higher risk of CVD and cardiovascular mortality [113,114,115].

In HMA studies, individuals with CVD most often demonstrate higher Pb concentrations than healthy controls. In a classic study from Pakistan, in patients after myocardial infarction, the mean hair Pb level was approximately twice as high as in healthy individuals (significant differences in both sexes) [104] Similarly, Ilyas et al. reported significantly higher Pb in patients with coronary heart disease, and an international analysis (Skalny et al., confirmed elevated Pb in patients with CHD, the highest in obese patients [105,106]. Although differences were smaller in populations with very low exposure, the overall picture is consistent with greater Pb accumulation in high-risk patients. The association between Pb and hypertension has been documented since the 1970s and is supported by more recent work, including mechanistic studies and cohorts from countries with low environmental exposure [116,117]. Clinically, lead increases the risk of hypertension and cardiovascular events through oxidative stress, inflammation, and endothelial dysfunction. Reviews and meta-analyses have shown significant associations, and declining renal filtration may mediate part of this effect. Furthermore, Pb accumulates in bones and may be released from them with age, sustaining exposure later in life [113,118].

HMA is a useful tool to characterize chronic lead exposure in environmental and occupational studies, and higher hair Pb has been associated with an adverse cardiovascular risk profile [77].

### 4.6. Magnesium

Most studies involving cardiac patients indicate lower magnesium concentrations in hair compared to those without coronary lesions. In the study by Urbanowicz et al., in patients with angiographically confirmed CAD, the median values were ~17 mg/kg vs. ~32 mg/kg in the group with normal arteries (*p* < 0.01), and the number of affected vessels correlated negatively with hair Mg levels (r = −0.237; *p* = 0.003) [119]. A similar trend was observed in metabolically challenged populations: in women with obesity and/or hypertension, hair Mg concentrations were significantly lower than in healthy individuals [91] “Neurological” data are less clear—a study of patients after acute ischemic stroke did not confirm differences in hair Mg levels compared to controls, highlighting the heterogeneity of HMAs depending on the individual and exposure time [120].

Mechanistically, Mg stabilizes cardiomyocyte and endothelial function, has vasodilatory, anti-inflammatory, and antiarrhythmic effects, and antagonizes Ca^2+^ (Mg deficiency increases Ca^2+^ influx, vasoconstriction, and blood pressure). Reviews indicate a role for disturbances in Mg homeostasis in the pathogenesis of hypertension and CVD [121]. At the level of systemic biomarkers, observations are more consistent than in HMA: in genetic studies (MR), higher genetically determined serum Mg concentration was associated with a lower risk of CAD, supporting a possible causal relationship; long-term cohort observations also link low Mg with a higher risk of cardiovascular events [122,123].

HMA magnesium can reflect long-term Mg status and has been associated with a greater atherosclerotic burden in CAD and in metabolically challenged populations, but its interpretation must consider diet, diabetes and diuretic use. In practice, serum or erythrocyte Mg is preferred for clinical decisions, while hair Mg is mainly informative in population studies; lower Mg (both systemic and in hair) generally corresponds to higher cardiovascular risk and provides a rationale for targeted correction of deficiency [34,119].

### 4.7. Calcium

In HMA studies, cardiac patients are more likely to have lower hair calcium concentrations than those with patent arteries; in the pilot study by Urbanowicz, the median Ca was ~100 mg/kg in CAD patients versus ~295 mg/kg in controls (*p* = 0.007), and the number of involved vessels showed a negative correlation with Ca levels (r = −0.217; *p* = 0.007) [119,124]. This relationship appears to be nonlinear—in the analysis including grading of coronary lesions, significant associations were found for single- and two-vessel disease, but disappeared in three-vessel disease (“all-or-nothing”), suggesting different dynamics in terminally advanced atherosclerosis [119]. The “deficient” Ca trend was also observed in metabolically challenged populations: in women with obesity and/or hypertension, hair Ca (and Mg) concentrations were significantly lower compared to healthy controls, which supports the association of Ca with the cardiovascular risk phenotype [91]. Mechanistically, calcium plays a dual role, serving as both an essential second messenger for contractility and conduction. However, chronic dysregulation of Ca^2+^ homeostasis promotes endothelial dysfunction, vascular remodeling, and calcification. Lower hair Calcium Levels may reflect long-term calcium redistribution or deficiency in soft tissues, with concomitant mineralization of the vascular wall [124]. At the intervention level, the data are equivocal: meta-analyses of RCTs signal a possible increase in CVD risk with Ca supplementation (especially in postmenopausal women), while reviews and analyses (including Cochrane) indicate a slight, statistically significant reduction in blood pressure with increasing Ca supply in people with low baseline intake, which highlights the importance of dietary context rather than routine “blind” supplementation [36,38,125]. A calcium-specific challenge in HMA is the high susceptibility of hair Ca measurements to external contamination from hard water and cosmetic products, as well as the influence of Ca/Mg balance. Therefore, hair calcium should be interpreted mainly as a long-term, auxiliary marker—particularly in relation to Ca/Mg ratios and in population studies—rather than as a standalone diagnostic tool. In clinical practice, management of calcium-related disturbances in CVD is still guided by the calcium–phosphate–PTH–vitamin D axis and global cardiovascular risk assessment [77,96].

### 4.8. Iron

HMA results for iron in CVD are inconsistent. In a classic post-myocardial infarction study (Pakistan), hair Fe concentrations were lower in patients than in controls, which was interpreted as reflecting Fe deficiency and common anemia in cardiac patients. However, in a more recent analysis of patients with angiographically confirmed CAD, median Fe values were slightly higher than in those with normal arteries (a statistically significant difference). Furthermore, in an independent study of CAD patients, higher hair Fe was associated with a lower SII inflammatory index (SII) (of the order of a statistically significant negative correlation). In contrast, no such associations were observed in the group with normal angiograms. This suggests that hair Fe levels may reflect both deficiency (e.g., in the course of anemia of chronic disease) and accumulation associated with chronic atherosclerosis, with the direction of change depending on the disease phenotype, inflammation, and iron status [119,126,127].

Mechanistically, iron has a “double-edged” effect: deficiency limits oxygen transport and worsens exercise tolerance and the course of heart failure (in randomized trials, intravenous administration of iron carboxymaltose improves symptoms and performance), while excess iron increases oxidative stress (Fenton reaction), LDL oxidation, and vascular wall inflammation, which fits the historical “iron hypothesis” of atherosclerosis, today understood more nuanced. More recent reviews emphasize that the role of Fe depends on the pool (tissue vs. circulating), localization (e.g., macrophages in plaques), and inflammatory context, and that patients with HH (hemochromatosis) do not necessarily have a higher incidence of atherosclerosis, indicating a complex regulation of vascular Fe homeostasis [128,129]. From a marker perspective, hair iron may still provide useful information on long-term iron status and its relationship to inflammatory burden and plaque biology. However, agreement with systemic markers is often imperfect at the individual level. Consequently, in clinical practice, ferritin, transferrin saturation and haemoglobin remain the primary tools for iron management in CVD, while HMA Fe is mainly used in environmental and exploratory research [119].

### 4.9. Arsenic

Arsenic is a highly toxic environmental element, and chronic exposure—primarily through contaminated water—is associated with an increased risk of CVD. In HMA studies, cardiac patients are more likely to have higher As concentrations in hair than healthy individuals, especially in areas with high exposure: in a study from West Bengal, the mean As level in CAD patients was ~0.7 µg/g, several times higher than in controls (~0.2 µg/g; *p* < 0.01), indicating a significant arsenic burden in this population [130]. A similar trend was observed in the international analysis: individuals with CHD had significantly higher hair As levels, and the highest values were recorded in patients with concomitant obesity, suggesting an additive (or synergistic) effect of obesity and As on metal retention and vascular risk [105]. At the population level, systematic reviews and meta-analyses confirm that chronic arsenic exposure increases the risk of CVD (including hypertension, IHD/AMI, and stroke), also at drinking water concentrations below traditional thresholds, which reinforces the importance of environmental prevention [131,132,133].

Mechanistically, as it damages the endothelium, increases oxidative stress and inflammation, disrupts glucose signaling and metabolism (promoting insulin resistance and diabetes), increases platelet aggregation, and can induce vasospasm, these phenomena collectively promote atherosclerosis, hypertension, and cardiovascular complications [134]. This interaction with obesity may result from altered As distribution/retention in adipose tissue and increased low-grade inflammation, which further burdens the cardiovascular system [105].

HMA is a standard toxicological tool for assessing chronic arsenic exposure. Due to the high affinity of As for keratin, levels greater than 1.0 µg/g in hair are considered evidence of overexposure and are also helpful for monitoring the effectiveness of interventions (e.g., water source replacement → decrease in hair As levels). Interpretation, however, requires context, as HMA does not distinguish between sources (e.g., inorganic arsenic from water vs. less toxic organic forms from seafood). In low-exposure populations, the differences can be subtle. Nevertheless, at the population level, high As in hair/nails correlates with a higher CVD burden and is helpful for environmental surveillance [135].

### 4.10. Mercury

Mercury (primarily methylmercury from marine fish) is associated with cardiovascular risk; however, the magnitude of this effect depends on population exposure levels and dietary co-occurrences. In a prospective study of middle-aged Finnish men, the highest quartiles of hair mercury (~2.4 µg/g) were associated with a significantly higher risk of myocardial infarction and cardiovascular death. Furthermore, high Hg accumulation attenuated the cardioprotective effect of fish consumption, suggesting an adverse interaction between Hg and n-3 PUFAs [136]. In contrast, two large US cohorts (Hg assessment in fingernails) showed no increased risk of CAD at lower exposure levels, highlighting the importance of geographic and exposure differences [137,138]. Populations with very high consumption of predatory fish (Amazonia) tend to have elevated hair mercury levels, and higher values correlate with a less favorable lipid profile (e.g., higher ApoB/ApoA1) and a higher estimated coronary risk [138]. Data syntheses indicate that Me-Hg exposure may increase the risk of IHD/stroke in dose–response analyses, although heterogeneity between studies remains significant [139].

Mechanistically, Hg increases oxidative stress (including by selenium capture and peroxidase inactivation), impairs endothelial function, increases blood pressure, and promotes arrhythmia; experimental observations also indicate accelerated atherosclerosis with chronic exposure [140]. In a population setting, the benefits of n-3 PUFAs may offset the harm associated with moderate Hg exposure. At the same time, at high concentrations (as in some Finnish studies), the protective effect of fish may be negated [136]. Selected NHANES analyses and other epidemiological studies link exposure to heavy metals, including Hg, with a higher risk of hypertension and CVD. However, the strength of the association for Hg tends to be weaker than for Pb/Cd and depends on the level of exposure [141,142].

HMA of mercury is a recognized method for assessing chronic exposure to methylmercury. Values greater than 1.0 µg/g (US EPA)–2.2 µg/g (WHO) in hair are considered elevated and potentially harmful, especially in women planning a pregnancy/pregnant women [143,144]. In environmental studies, HMA can help monitor interventions (e.g., changing water source/fish selection) and predict population risk; however, limitations include the specificity of the biomarker (hair primarily reflects methylmercury from fish, less so inorganic forms), the time horizon of several months, and the influence of dietary patterns [136].

### 4.11. Chromium

HMA data in CVD indicate that hair Cr levels are sometimes elevated in patients with coronary artery disease. In a Pakistani study, post-infarction patients had significantly higher mean hair Cr concentrations than controls, which the authors attributed to dietary specificity and possible compensation for metabolic disorders [106]. Similar results were obtained in the study by Urbanowicz: patients with CAD had higher Cr levels than those with normal arteries (*p* < 0.001), and the number of affected vessels positively correlated with Cr (r ≈ 0.24); interestingly, the effect disappeared in patients with three-vessel disease, which the authors termed the “all-or-nothing” phenomenon [119]. Additionally, in patients with CAD, a negative correlation was observed between hair Cr and the inflammatory index SII (r ≈ −0.48), which was not seen in the group with normal angiograms. This may suggest that higher Cr is associated with a lower inflammatory burden in this population [127,145,146,147].

Mechanistically, Cr(III) is a metabolically beneficial element in small amounts (modulating insulin signaling and glucose tolerance), which may explain the observation of higher Cr(III) in patients with metabolic disorders as a compensatory phenomenon; however, toxic Cr(VI) from environmental/industrial exposure causes oxidative stress and tissue damage, and reports also link it to cardiovascular risk [148]. In studies examining the distribution of atherosclerotic lesions, higher hair Cr levels were associated with the location and severity of lesions in individual coronary artery branches, supporting the hypothesis that Cr plays a role as a marker associated with the progression of CAD (although it does not determine the causal direction) [21].

Hair chromium reflects long-term Cr intake and exposure, but absolute levels are very low and may be influenced by environmental or industrial dust. Currently, HMA Cr is primarily confined to research settings, where associations with CAD severity and inflammatory indices (e.g., SII) are being investigated. Routine assessment of metabolic and inflammatory status in CVD still relies on systemic markers [119,149].

### 4.12. Manganese

Manganese, a key cofactor of mitochondrial superoxide dismutase (Mn-SOD), plays a crucial role in defense against oxidative stress and metabolic regulation; therefore, its deficiency may contribute to endothelial dysfunction and the progression of atherosclerosis. Current HMA data indicate that hair Mn concentrations are slightly but significantly lower in patients with angiographically confirmed CAD than in those with patent arteries (median values of 0.20 vs. 0.22 mg/kg; *p* < 0.001), suggesting that even subtle Mn depletions may have biological significance. A similar trend was observed in women with obesity and/or hypertension, whose hair Mn levels were significantly reduced compared to controls [91,119]. Not all populations confirm these differences (a study following myocardial infarction in Pakistan found Mn values “comparable” between patients and controls), which most likely reflects variations in diet and environmental exposure [106]. Mechanistically, a decrease in Mn availability may limit the activity of Mn-SOD—the only SOD located in the mitochondrial matrix—enhancing the formation of superoxide anion radicals and secondarily promoting oxidative stress, inflammation, and impaired vascular reactivity. Experimental and review data emphasize the central role of Mn-SOD in maintaining the mitochondrial integrity of cardiomyocytes [150,151]. From a biomarker standpoint, hair manganese primarily reflects long-term Mn intake and shows sensitivity to geographical and dietary patterns. In cardiovascular settings, clinical conclusions should still be based mainly on the assessment of nutritional status and dietary modification (e.g., whole grains, nuts), while HMA Mn serves as a research tool to identify mild Mn deficiency or excess in specific risk groups [152].

### 4.13. Lithium

HMA data indicate that the presence of coronary artery disease alone is not associated with changes in hair lithium content. In a study including patients with angiographically confirmed CAD and those with patent arteries, median hair Li levels remained very low and comparable (≈0.02 µg/g), suggesting no differences between groups at typical environmental exposure levels [119]. At the same time, in an independent cohort of CAD patients, a significant negative correlation was found between hair Li concentration and the Systemic Immune-Inflammation Index (SII)—this relationship was not observed in individuals with normal angiograms, which may indicate that higher long-term Li “status” coexists with a lower severity of the inflammatory response in this population [127]. Mechanistically, lithium (even in trace doses from diet and water) has been linked to modulation of inflammatory and neurohormonal pathways (including the sympathetic axis and GSK-3 cascades). Ecological observations suggest that regions with higher natural lithium content in water are characterized by more favorable population health indicators (e.g., lower suicide mortality). However, the results are heterogeneous and do not directly relate to cardiovascular risk [153]. From the perspective of HMA, lithium primarily reflects long-term environmental exposure, with hair levels strongly influenced by regional water content and often approaching the analytical limit of quantification. Currently, there is no indication for routine hair Li measurement in CVD risk stratification; its potential indirect role, via modulation of inflammation, remains a research question for future prospective studies combining hair, serum, and water measurements with hard cardiovascular endpoints [119,154].

### 4.14. Nickel

Nickel is a common environmental pollutant (metallurgical emissions, exhaust fumes, tobacco smoke) and has been linked to the progression of CVDs. HMA studies more frequently observe higher Ni concentrations in the hair of patients with cardiac conditions. In a classic study, Ni levels in post-MI patients were significantly higher than in the control group, which the authors interpreted as reflecting increased exposure/accumulation in individuals with MI [104]. These results correspond with a prospective analysis of a Polish cohort with angiographically confirmed CAD, in which median Ni levels in patients were higher than in those with patent arteries (however, the difference did not reach significance in a two-group comparison). In contrast, a positive correlation between Ni levels in hair and the extent of atherosclerotic lesions (number of affected vessels) indicates an association between Ni and disease progression (r ≈ 0.18; *p* ≈ 0.03) [119]. Further angiographic studies also suggest a localization dimension of this association: a relationship was found between the presence of plaques in the left main coronary artery and higher Ni content (as well as Zn and Sb) in hair, which may reflect a less favorable anatomical phenotype of the disease [119]. Beyond hair biomarkers, NHANES population analyses have shown that higher environmental exposure to nickel (measured by urinary Ni concentration) is associated with a higher risk of CVDs, independent of classical risk factors [155].

Mechanistically, nickel increases oxidative stress and lipid peroxidation, promoting endothelial dysfunction and inflammatory processes in the vascular wall; experimental reports and pathophysiological reviews place Ni within a broader spectrum of cardiotoxic metals with proatherogenic effects [152,156,157]. Furthermore, Ni may modulate the immune response (acting as a contact allergen) and influence ionic signaling, which together may promote plaque instability. Overall, the empirical and epidemiological data indicate that higher Ni exposure/accumulation is associated with a higher atherosclerotic burden and unfavorable lesion localization. However, the signal strength may be subtle in cross-sectional analyses [119].

From a marker perspective, hair nickel reflects chronic environmental exposure and is useful in population biomonitoring. Because Ni is ubiquitous and external contamination from metal dust and jewellery is common, interpretation of single-patient results requires particular caution. In clinical and research cohorts, excluding individuals with obvious occupational exposure increases the likelihood that hair Ni represents endogenous accumulation. Although HMA Ni is not a routine CVD risk test, it can prompt the identification and reduction of environmental and occupational sources of exposure when interpreted in conjunction with other cardiovascular risk factors [119].

### 4.15. Aluminum

Although it has no recognized biological role, aluminum is one of the most abundant metals in the environment. In recent years, increasing evidence suggests that its accumulation in the body may be associated with metabolic disorders and an increased risk of CVD. A study by Skalny et al. [105], using ICP-MS spectrometry, demonstrated that patients with CHD had significantly higher hair aluminum concentrations compared to healthy individuals, with the highest values observed in patients with comorbid obesity. These results suggest a synergistic effect of obesity and Al exposure, leading to increased retention of this element. Similar observations were reported in population studies unrelated to occupational exposure—overweight and obese individuals had significantly higher concentrations of aluminum in hair and urine, indicating a potential role of aluminum in the pathogenesis of metabolic syndrome [91].

The mechanisms of aluminum toxicity in the cardiovascular system are multifactorial. As indicated by Tinkov et al. (2019), chronic exposure Al may lead to increased oxidative stress, disturbances in calcium-phosphate homeostasis, endothelial dysfunction, and vascular remodeling, promoting the development of atherosclerosis [158]. Increased generation of reactive oxygen species may induce lipid peroxidation and activate pro-inflammatory pathways in the vessel wall. Moreover, aluminum binds to phosphates, disrupting calcium metabolism and parathyroid function, which, in the long term, may lead to vascular calcification and cardiac dysfunction. In dialysis patients exposed to long-term contact with aluminum contained in dialysis fluids, the so-called aluminum cardiomyopathy has been described, manifested by myocardial hypertrophy and impaired ejection fraction [15,159]. Drinking water remains a significant source of environmental exposure to aluminum. The coagulation process using aluminum salts may result in the presence of residual Al in treated water. This level depends on the chemical speciation of the coagulant, pH, and the quality of the filtration. The WHO emphasizes that long-term consumption of water containing more than 0.2 mg/L Al may pose a health risk factor, especially in individuals with kidney disease [160,161]. Modern studies have shown that changing water treatment methods and controlling process parameters can reduce Al content. Consequently, hair is used in assessing exposure to aluminum, for example, in industrial workers (Such as those in foundries) and in populations with contaminated water. It has been shown that Al concentration in hair decreases after providing cleaner drinking water [160]. Hair aluminium has therefore proven most useful in comparative contexts (group-to-group or before-and-after environmental interventions), where relative differences are more informative than absolute cut-offs. Elevated hair Al suggests increased environmental exposure, which—as current data indicate—may act synergistically with factors such as obesity and smoking to worsen cardiometabolic risk. In cardiovascular patients, it appears reasonable to minimize avoidable Al exposure (e.g., from certain water sources, cookware, cosmetics), although the direct impact of such measures on hard CVD outcomes has not yet been formally established [91].

## 5. Practical Implications and Research Gaps

HMA can meaningfully complement current cardiovascular risk assessment algorithms only in precisely defined clinical and epidemiological situations, where typical short-term markers (blood, urine) fail to assess the burden of multi-month environmental exposure. The most justified application area is populations with high, chronic exposure to toxic metals (e.g., Pb, Cd, As, Hg, Al, Ni)—residents of industrial or mining areas, or those with water quality problems—where HMA can act as a “long tape” of exposure and identify individuals for whom it is worthwhile to implement environmental interventions (changing water sources, limiting occupational exposure) and nutritional interventions (modifying the consumption of fish with high Hg content, balancing the supply of protective micronutrients) alongside classic risk assessment. This is confirmed by numerous reviews linking metal exposure to CVD risk and studies emphasizing the role of aluminum/arsenic as proatherogenic factors [15,156,162,163].

In primary prevention, especially in individuals with a metabolic risk phenotype (obesity, insulin resistance), HMA can broaden the picture to include an “elemental profile”—deficiencies of protective elements (e.g., Mg, Mn, Se) and the accumulation of biologically irrelevant metals—which enhance oxidative stress and inflammation and are not accounted for by classic risk calculators (e.g., SCORE2/SCORE2-OP). However, intervention decisions must be based on validated cardiac models, as these determine population prognosis [164].

Currently, the evidence is insufficient to recommend HMA for routine, individual risk stratification. The main limitations include the predominance of cross-sectional studies over prospective studies with hard endpoints, heterogeneity of matrices and methods (site and length of collection, washing procedures, digestion, ICP-MS/ICP-OES technique), sensitivity to external contamination, and the lack of agreed-upon reporting standards—these factors limit the comparability of results between centers and prevent the calibration of “norms” for CVD [77,96]. Translation from the level of environment-CVD associations (well documented for several metals, including As, Pb, Cd, Ni) to the level of reliable clinical prediction is necessary, demonstrating the added value of HMA over established models (e.g., SCORE2)—which requires prospective studies with parallel measurements of elements in hair, blood, and urine and with assessment of the incremental predictive power [154,165,166].

Research priorities should include: (1) multicenter, prospective cohorts with predefined, hard CVD endpoints that map dose–response relationships between quantiles of hair metal concentrations (and key ratios, e.g., Zn/Cu, Ca/Mg, Se/Hg) and cardiac events; (2) full preanalytical and analytical standardization of HMAs (refined collection protocols, validated decontamination and digestion regimens, QA/QC, use of ICP-MS/LA-ICP-MS), along with public, stratified reference value databases stratified by sex, age, region, and exposure profile; (3) integrative analyses in which the HMA panel is added to SCORE2/SCORE2-OP models and tested for clinical utility (reclassification of patients in borderline zones, impact on decisions to intensify pharmacological or environmental prevention) [96,164]. In parallel, it would be worthwhile to develop “bio-verified” environmental interventions—for example, programs to improve water quality or reduce industrial emissions, accompanied by monitoring of hair metal load decline—as a pragmatic test of risk reversibility in high-exposure populations [162,164,167]

Until these conditions are met, HMA should be considered a complementary tool for niche applications (documented, chronic environmental/occupational exposure; population studies and evaluation of intervention effectiveness) rather than a standalone element of clinical risk stratification. Treatment decisions should be based primarily on guidelines and validated calculators. If HMA results are used, they should be interpreted in the strict context of the patient’s exposure, diet, and metabolic phenotype [156].

## 6. Conclusions

The collected data indicate that HMA reflects long-term environmental and dietary exposure to metals and trace elements, revealing patterns potentially relevant to the pathogenesis of CVDs. Within the toxic heavy metal groups, the most consistent signals concern cadmium and lead (often higher levels in patients, associated with hypertension and atherosclerosis) and arsenic (highly toxic, with apparent differences in populations with high exposure). Mercury exhibits context dependence: with high consumption of predatory fish, its increase may negate some of the cardioprotective benefits of a fish diet, while with lower exposure, the effect may be neutral. Among common environmental metals, nickel and aluminum are more frequently associated with an unfavorable metabolic phenotype (obesity, insulin resistance) and a higher atherosclerotic burden, although causality remains unclear. Among the “protective” elements, HMA more often reveals lower magnesium and manganese levels in patients, consistent with their role in controlling oxidative stress and vascular reactivity; hair calcium tends to be reduced in CAD in a nonlinear manner (possible vascular distribution and calcification), while iron and chromium reveal heterogeneous results (from deficiency signals to excess/accumulation in specific phenotypes, with significant dependencies on inflammation and metabolism). Copper remains ambiguous (Zn/Cu as an important indicator of redox balance). At the same time, lithium—at trace levels—does not consistently differentiate between patients and healthy individuals, although it may modulate inflammatory signals. Clinically, HMA does not replace validated risk calculators (SCORE2/PCE). However, it can complement assessment in individuals with chronic, documented environmental exposures (such as water, occupation, or industrial areas) and in the primary prevention of high-metabolic-risk phenotypes, where environmental “imprints” accumulate over time and contribute to oxidative stress and inflammation. In these scenarios, HMA helps identify modifiable sources of exposure (e.g., dietary arsenic/mercury, environmental lead/cadmium/nickel) and protective micronutrient deficiencies (Mg, Mn, Zn/Se), guiding environmental and nutritional interventions. However, evidence is insufficient to use HMA for routine, individual risk stratification, as cross-sectional studies predominate, analytical procedures are inconsistent (due to variations in collection, decontamination, and ICP-MS/ICP-OES), and results are susceptible to contamination and regional/dietary influences. Prospective cohorts with parallel measurements in hair/blood/urine and hard CVD endpoints, pre- and analytical standardization, and the development of CVD-specific reference thresholds—both absolute values (e.g., Pb, Cd, As, Hg, Ni, Al) and balance indices (Zn/Cu, Ca/Mg, Se/Hg)—tested for their added value over clinical models (ΔC-statistic, NRI/IDI) remain a priority. Until such data are available, HMA should be used selectively (in environmental/occupational exposure, population-based surveillance, and assessment of intervention efficacy) and its results interpreted within the strict clinical, nutritional, and environmental context of the patient.

## Figures and Tables

**Table 1:** The table presents key macro elements relevant to cardiovascular physiology and disease, outlining their biological roles, mechanistic importance, and clinical implications. (↑—increase, →—no suggested role).

Element	Physiological Role	Main CVD-Related Effects		**References**
		Deficiency	Excess	
Sodium (Na)	Central extracellular cation; regulates fluid volume, osmotic pressure and neuromuscular excitability.	water retention, ↑ circulating blood volume, activation of RAAS and sympathetic nervous system, endothelial dysfunction and ↑ arterial stiffness → ↑ blood pressure, ↑ risk of stroke, coronary artery disease and heart failure.	usually secondary to HF or intensive diuretic therapy; strong marker of poor prognosis in heart failure.	[27,28,29]
Potassium (K)	Principal intracellular cation; determines proper repolarisation of cardiomyocytes, has vasodilatory effects and promotes natriuresis.	prolongs repolarisation and QT interval, ↑ risk of ventricular arrhythmias, especially during diuretic therapy.	disturbs atrioventricular conduction (bradycardia, AV block, asystole), typically in CKD or during ACEI/ARB/MRA therapy.	[30,31]
Magnesium (Mg)	Cofactor of >300 enzymes; “natural antagonist” of calcium; stabilises membrane potential and affects vascular tone and conductivity.	QT prolongation and torsade de pointes, ↑ risk of atrial fibrillation, difficult correction of hypokalaemia, association with ↑ blood pressure and stroke risk.	rare outside CKD or rapid i.v. MgSO_4_; may cause hypotension and bradycardia.	[32,33,34]
Calcium (Ca)	Second messenger crucial for cardiomyocyte contraction; regulated by PTH, calcitriol and calcitonin; important for coagulation and mineral metabolism.	QT prolongation, possible impairment of contractility; secondary hyperparathyroidism promotes proatherogenic processes.	QT shortening, risk of arrhythmias; chronically (particularly with hyperphosphataemia) promotes vascular and valvular calcification.	[35,36,37,38]
Phosphorus (P)	Structural component of hydroxyapatite, key element of ATP, component of cell membranes (phospholipids).	-	induces osteochondrogenic transformation of vascular smooth muscle cells → calcification of arterial walls and aortic valve, ↑ arterial stiffness and cardiovascular risk.	[39,40,41]

**Table 2:** The table presents key microelements relevant to cardiovascular physiology and disease, outlining their biological roles, mechanistic importance, and clinical implications. (↑—increase, →—no suggested role, ↓—decrease).

Element	Physiological Role	Main CVD-Related Effects		References
		Deficiency	Excess	
Zinc (Zn)	Cofactor of many enzymes (incl. Cu/Zn-SOD); supports redox balance, immunity and endothelial NO.	↑ oxidative stress and inflammation, endothelial dysfunction, atherogenic lipid profile → ↑ risk of CHD and HF.	Chronic high-dose supplements → secondary Cu deficiency (anaemia, neutropenia, possible adverse CV impact).	[42,43,44]
Selenium (Se)	Component of selenoproteins (GPx, TrxR, deiodinases); crucial for antioxidant defence and thyroid function.	Endothelial and LDL oxidative damage, impaired myocardial protection (e.g., Keshan cardiomyopathy), ↑ risk of CHD/HF/stroke.	Selenosis; U-shaped association—very high Se levels provide no extra benefit and may be linked to adverse outcomes.	[17,45,46,47,48]
Iron (Fe)	Heme and Fe–S cluster component; essential for mitochondrial respiration and oxygen transport.	Very common in HF → ↓ exercise capacity, ↑ hospitalisations and mortality; i.v. iron improves symptoms and HF outcomes when deficiency is present.	Iron overload (e.g., haemochromatosis, transfusions) → cardiomyopathy, arrhythmias via oxidative stress and ferroptosis.	[49,50,51,52,53,54]
Copper (Cu)	Cofactor of oxidases (cytochrome c oxidase, Cu/Zn-SOD, lysyl oxidase); involved in Fe metabolism and vascular matrix integrity.	Fe-resistant anaemia, endothelial dysfunction, weakened vascular connective tissue, possible ↑ BP and adverse lipid changes.	↑ plasma Cu and high Cu:Zn ratio mark inflammation and are associated with ↑ CV morbidity and mortality.	[55,56,57,58]
Manganese (Mn)	Cofactor of mitochondrial Mn-SOD and several metabolic enzymes; supports mitochondrial antioxidant defence.	Theoretically ↑ mitochondrial ROS and endothelial dysfunction, potentially favouring atherosclerosis (clinically rare).	Mainly neurotoxic; with normal renal function, clinically relevant cardiac effects are uncommon.	[59,60]
Chromium (Cr)	Modulates insulin signalling and glucose tolerance; indirectly affects lipids and low-grade inflammation	Impaired glucose tolerance/insulin resistance → indirectly ↑ CVD risk.	Nutritional supplementation: modest improvements in glycaemia/lipids; no clear evidence of reduced major CV events without true deficiency; high occupational Cr(VI) exposure is toxic and may promote CV damage.	[61,62]
Iodine (I)	Required for thyroid hormone (T3/T4) synthesis; T3/T4 regulate HR, contractility, SVR and lipid metabolism.	Hypothyroidism → bradycardia, exercise intolerance, ↑ LDL and atherogenic profile → ↑ atherosclerosis risk.	Hyperthyroidism (often iodine-related) → tachyarrhythmias (especially AF), ↑ cardiac output and myocardial oxygen demand.	[63,64]

## Data Availability

This study is a review article and does not report any new data. All data discussed in this manuscript are available in the cited primary sources and published literature.

## Supplementary Materials

The following supporting information can be downloaded at: https://www.mdpi.com/article/10.3390/ijms262412145/s1.
