# Supplementary material for: Essential and Toxic Elements in Cardiovascular Disease: Pathophysiological Roles and the Emerging Contribution of Hair Mineral Analysis
Source: Int J Mol Sci. 2025 Dec 17;26(24):12145. doi: 10.3390/ijms262412145 (PMC12733699; doi:10.3390/ijms262412145)
Supplement: Supplementary file 1 [file ijms-26-12145-s001.zip › ijms-3998606-supplementary materials.pdf]

Supplementary Materials Table S1. Clinically relevant drug–macronutrient interactions: agents modifying levels, key cardiologic pharmacodynamic effects and practical monitoring notes.[based on https://go.drugbank.com/food-interaction-checker]

Element	Drug ↑ concentration/retention	Drug ↓ concentration / increasing loss	Pharmacodynamic interactions (cardiologically significant)	Monitoring / Practical Notes
**Sodium (Na)**	NSAIDs, glucocorticoids, mineralocorticoids (fludrocortisone), some CCBs (excess Na exacerbates edema), sodium preparations of drugs and infusions; low Na in the diet ↓ affects the effectiveness of these effects	Loop/thiazide diuretics, SI-ADH-inducing (SSRI, carbamazepine), desmopressin	A high-sodium diet reduces the effectiveness of antihypertensive drugs (especially diuretics and RAS inhibitors); large fluctuations in sodium affect blood volume and heart rate.	In patients with HT/HF: recommend a low-sodium diet; consider a “salt test” in resistant HT; remember that sodium affects lithium clearance (high Na → ↓ lithium concentrations; low Na → ↑ lithium toxicity)
**Potassium (K)**	ACEI/ARB, MRA (spironolactone/eplerenone), amiloride, triamterene, heparin, cyclosporine/tacrolimus, TMP-SMX, NSAIDs, aliskiren, sacubitril/valsartan	Loop/thiazide diuretics, glucocorticosteroids, beta2-agonists (salbutamol), insulin + glucose (cell shift), theophylline, amphotericin B, high doses of ion exchange resins (polystyrene sulfonate)	Hypokalemia → increases digoxin toxicity and proarrhythmic effects of QT-prolonging drugs (so-talol, dofetilide); hyperkalemia → conduction disturbances, asystole, especially with RAA block.	Routinely monitor potassium after initiation/dose change of ACEI/ARB/MRA or diuretic; avoid potassium salt in CKD; target in cardiac patients usually 4.0–5.0 mmol/l.
**Magnesium (Mg)**	Intravenous administration, Mg salts; rarely hypermagnesemia in CKD or excess of Mg medications (laxatives, antiacids)	Loop/thiazide diuretics, PPIs (chronic), amphotericin B, aminoglycosides, cis-platin, cyclosporine/tacrolimus, alcohol	Hypomagnesemia → torsade de pointes, atrial fibrillation; increases hypokalemia resistant to supplementation; Mg i.v. is the first-line drug in torsade de pointes	Check Mg in case of arrhythmias, use of PPIs/diuretics; correct Mg in parallel with K.
**Calcium (Ca)**	Thiazides (hypercalcemia), vitamin D, Ca preparations, lithium (may ↑ Ca), immobilization	Loop diuretics (hypocalcemia), calcitonin, bisphosphonates, denosumab, glucocorticosteroids	Hypercalcemia increases digoxin toxicity; shortens QT (arrhythmias); Ca i.v. antagonizes CCB overdose (detoxification therapy)	Separate from levothyroxine, tetracyclines, fluoroquinolones, bisphosphonates (≥2–4 h); consider caution with Ca supplements in individuals at high risk of CVD

**Phosphorus (P)**	In CKD: retention (hyperphosphatemia)	Loop diuretics (P loss), phosphate binders (sevelamer, lanthanum carbonate)	Hyperphosphatemia + Ca → vascular/valve calcification; increased arterial stiffness	In patients with CKD, limit phosphates (especially those found in food additives); consider using nephrophosphate binders.

Supplementary Materials Table S2. The table summarizes clinically relevant drug-micronutrient interactions [based on https://go.drugbank.com/food-interaction-checker]

Element	Drug ↑ concentration/retention	Drug ↓ concentration / increasing loss	Pharmacodynamic interactions (cardiologically significant)	Monitoring / Practical Notes
**Iron (Fe)**	-	IPP/antacid, calcium carbonate, high doses of Ca; tetracyclines, fluoroquinolones (bilateral chelation); foods with phytates/polyphenols	Fe deficiency worsens performance in HF; excess (hemochromatosis) → cardiomyopathy/arrhythmias (oxidative stress)	Separate Fe from levothyroxine, tetracyclines, fluoroquinolones (≥2–4 h), and from coffee/tea; in HF, prefer i.v. If deficiency is present, monitor ferritin/TSAT.
**Cynk (Zn)**	-	Long-term: thiazides/loop and ACEI ↑ Zn loss; chelators (penicillamine)	High doses of Zn → copper (Cu) deficiency (anemia, neutropenia), which may secondarily harm the CV; Zn deficiency promotes endothelial dysfunction	Separate Zn from tetracyclines/fluoroquinolones (chelation); long-term Zn supplementation >40 mg/d → consider Cu 1–2 mg/d and/or Cu control

**Selenium (Se)**	—	—<br>(good bioavailability; no typical drugs significantly ↓ affect Se)	No strong drug-drug interactions; indirect significance via thyroid (deiodinases) and redox	Avoid exceeding 400 μg/d; supplementation is not routinely required without deficiency
**Copper (Cu)**	Estrogens/pregnancy ↑ ceruloplasmin (↑ total Cu)	High doses of Zn (competition), penicillamine/trientine (chelation), and rarely thiazides	Cu deficiency → Fe-resistant anemia, elastin weakening (theoretically aneurysms/HT)	With long-term Zn supplementation, monitor Cu deficiency symptoms, including anemia, neutropenia, and sensory loss.
**Manganese (Mn)**	Cholestasis/CKD (retention)	Large doses of Fe (competition for absorption)	No significant cardiac interactions; mainly neurotoxicity	Supplementation is rarely necessary; no routine interactions with cardiovascular medications
**Chromium (Cr)**	—	GKS and some β-blockers may ↑ cause Cr loss; phytic acids ↓ absorption	Cr supplements may potentiate the effects of insulin/oral antidiabetic drugs (hypoglycemia)	Consider caution when treating diabetes; no direct interactions with common cardiovascular medications

**Iodine (I)**	Amiodarone, iodinated contrast agents (high iodine loads)	—	Thyroid disorders (hyperthyroidism/hypothyroidism) alter rhythm/contractility and lipid profile (e.g., AF in hyperthyroidism)	In patients on amiodarone, monitor TSH/T4/T3; diet: standard salt iodization is usually sufficien
